# Genetic Architecture of Highly Complex Chemical Resistance Traits across Four Yeast Strains

**DOI:** 10.1371/journal.pgen.1002570

**Published:** 2012-03-15

**Authors:** Ian M. Ehrenreich, Joshua Bloom, Noorossadat Torabi, Xin Wang, Yue Jia, Leonid Kruglyak

**Affiliations:** 1 Lewis-Sigler Institute for Integrative Genomics, Princeton University, Princeton, New Jersey, United States of America; 2 Department of Ecology and Evolutionary Biology, Princeton University, Princeton, New Jersey, United States of America; 3 Howard Hughes Medical Institute, Princeton University, Princeton, New Jersey, United States of America; 4 Molecular and Computational Biology Section, University of Southern California, Los Angeles, California, United States of America; 5 Department of Molecular Biology, Princeton University, Princeton, New Jersey, United States of America; University of Wisconsin-Madison, United States of America

## Abstract

Many questions about the genetic basis of complex traits remain unanswered. This is in part due to the low statistical power of traditional genetic mapping studies. We used a statistically powerful approach, extreme QTL mapping (X-QTL), to identify the genetic basis of resistance to 13 chemicals in all 6 pairwise crosses of four ecologically and genetically diverse yeast strains, and we detected a total of more than 800 loci. We found that the number of loci detected in each experiment was primarily a function of the trait (explaining 46% of the variance) rather than the cross (11%), suggesting that the level of genetic complexity is a consistent property of a trait across different genetic backgrounds. Further, we observed that most loci had trait-specific effects, although a small number of loci with effects in many conditions were identified. We used the patterns of resistance and susceptibility alleles in the four parent strains to make inferences about the allele frequency spectrum of functional variants. We also observed evidence of more complex allelic series at a number of loci, as well as strain-specific signatures of selection. These results improve our understanding of complex traits in yeast and have implications for study design in other organisms.

## Author Summary

Most heritable traits of agricultural, evolutionary, and medical significance are specified by multiple genetic loci. Despite decades of research, we have only a limited understanding of the genetic basis of such complex traits. Studies in model organisms have the potential to provide fundamental insights into this research area, but most genetic mapping studies in these species have had low statistical power to detect multiple loci with small effects. Using a technique in which we employed millions of cross progeny in genetic mapping, we previously showed that resistance to chemicals has a highly complex genetic basis in a cross of a lab strain and a wine strain of the budding yeast *Saccharomyces cerevisiae*. Because we only examined a single cross, it was unclear how general our findings were. Here, we expand our work to all six possible crosses of four strains—the two isolates we used in our last study, as well as an isolate from an immunocompromised human being and an isolate from the sap of an oak tree. Our results based on these four ecologically and genetically distinct *S. cerevisiae* strains suggest that resistance to chemicals commonly exhibits a highly complex genetic basis among yeast isolates.

## Introduction

Most traits of agricultural, evolutionary, and medical significance are genetically complex, involving multiple genes that interact with one another and the environment [1]. Despite decades of effort, our understanding of how such traits are specified at the genetic level remains incomplete [2]. Studies in model organisms can provide fundamental insights into the genetic basis of complex traits that are applicable to other species, including humans [3]. However, such studies typically detect only a small fraction of the loci that contribute to a trait due to low statistical power [4].

To improve genetic mapping of complex traits in *Saccharomyces cerevisiae*, we recently developed extreme QTL mapping (X-QTL), which is a bulk segregant mapping technique that employs millions of cross progeny [5]. X-QTL involves three key steps: generation of very large segregating populations, isolation of cross progeny with extreme trait values, and quantitative measurement of pooled allele frequencies across the genome in these phenotypically extreme individuals [5]. To make the pools of segregants that are the starting point for X-QTL, we use selectable markers to obtain an effectively unlimited number of progeny from a cross of two strains. We then employ selection-based phenotyping to isolate large numbers of segregants with extreme trait values from populations that contain millions of cross progeny. DNA is extracted from pools of phenotypically extreme segregants, and the allele frequencies of markers throughout these individuals' genomes are determined using custom microarrays or next generation sequencing. In an X-QTL experiment, a locus that influences a trait is expected to show an allele frequency skew in the direction of the parental allele that contributes to a more extreme trait value.

By applying X-QTL to a number of chemical resistance phenotypes in a single cross of the lab strain BY4716 and the vineyard strain RM11-1a (hereafter, BY and RM, respectively), we were able to show that large numbers of loci can underlie quantitative trait variation between *S. cerevisiae* isolates [5]. Following our publication, another group observed similar results in a different cross [6], suggesting that high genetic complexity may be a common feature of heritable trait variation among yeast strains.

Here, we examined how genetic complexity varies among strains and crosses. We used X-QTL to identify the genetic basis of resistance to 13 diverse chemicals in all 6 pairwise crosses of strains BY, RM, YJM789, and YPS163. YJM789 (hereafter, YJM) is derived from a clinical isolate, and YPS163 (hereafter, YPS) is an oak strain. These 4 strains are highly diverged at the sequence level [7], [8], [9], [10], [11] and exhibit a wide range of heritable phenotypic differences [12], [13], [14], [15], [16], [17], [18], [19]. Because of the statistical power gained by using very large mapping populations, we detected approximately an order of magnitude more loci than did previous studies involving multiple crosses of yeast strains [15], [17], [20], allowing us to gain deeper insights into the genetic architecture and evolution of complex traits in *S. cerevisiae*.

## Results/Discussion

We previously noted that levels of genetic complexity underlying heritable variation in growth differed among chemical conditions in a single cross [5]. Here, we sought to determine the generality of our previous finding by examining additional crosses. We first generated the strains and microarrays to conduct X-QTL in all 6 pairwise crosses of the BY, RM, YJM, and YPS strains (Materials and Methods). Because the statistical power of X-QTL is dependent on effective enrichment of highly resistant cross progeny in a segregating pool, and the crosses vary in their genetic compositions, leading to different distributions of resistance among the progeny of each cross, we used dose-response experiments to determine cross-specific, highly selective drug concentrations for each of 13 diverse chemicals that resulted in similar selection intensities for all crosses (Materials and Methods; File S1). Once the selective doses were determined, we conducted one X-QTL experiment for each chemical and cross combination.

**Figure 1:**
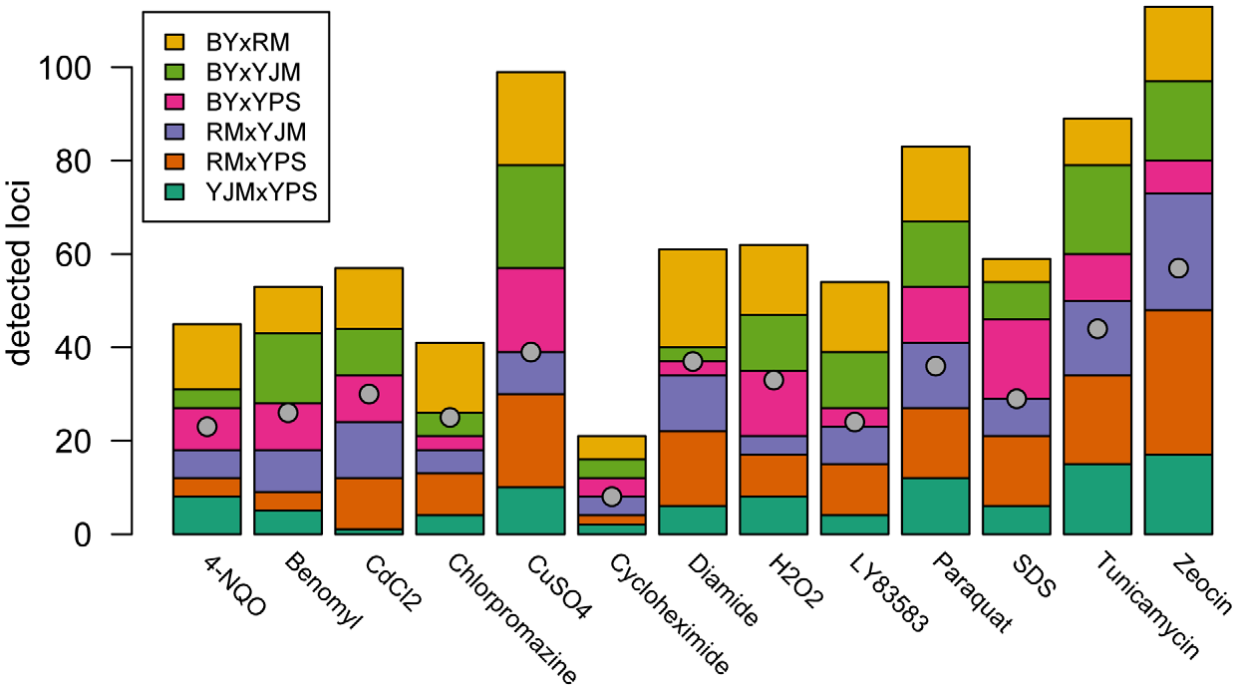
Numbers of detected peaks and distinct loci. The stacked bar plots show the number of peaks detected for each trait using X-QTL selections in each cross. The first parent listed in each cross was MATα and the second parent was MAT**a**. The grey dots indicate the number of distinct loci detected in a condition after peak grouping.

We observed substantial variation in the number of loci detected in different conditions and crosses (Figure 1). Across all 78 X-QTL experiments, we identified 837 total peaks at a False Discovery Rate (FDR) of 1%, or an average of 10.7 peaks per trait per cross (Figure 1; Figure S1A–S1M). Both the chemical and the cross had significant effects on the number of peaks detected in an X-QTL experiment (ANOVA, chemical effect F = 5.27, d.f. = 12, p = 5.67×10^−6^; cross effect F = 3.14, d.f. = 5, p = 0.014), with the effect of the chemical (partial R^2^ = 0.46) being much larger than the effect of the cross (partial R^2^ = 0.11). An ANOVA testing the effects of chemical and strain resulted in a similar effect of chemical on the number of detected peaks (partial R^2^ = 0.46; F = 4.52, d.f. = 12, p = 3.51×10^−5^), but no strain had a significant effect on its own (partial R^2^<0.02; F<2.5, d.f. = 1, p>0.12; Materials and Methods). Consistent with a comparatively small effect of strain background on genetic complexity, only one trait showed a significant excess of peaks in crosses involving any one strain: crosses in which RM was one of the parents had an excess of peaks in diamide (χ2 = 22.44, d.f. = 1, Bonferroni-corrected p = 1.97×10^−4^; Figure 1). These results suggest that genetic complexity in yeast is mainly a property of the trait being examined rather than of the strain background.

**Figure 2:**
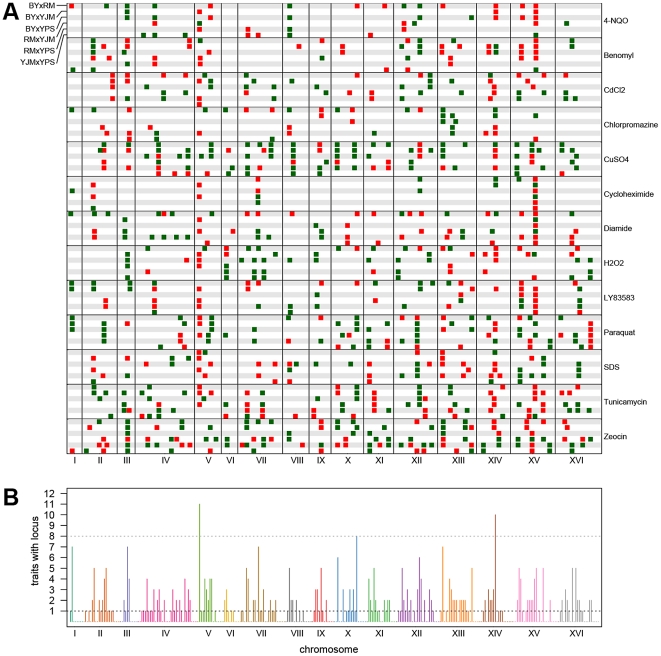
Genome-wide plots of detected loci. (A) Loci detected for each cross and trait, with green indicating loci selected in the direction of the MATα parent and red indicating loci selected in the direction of the MATa parent. For each trait, the crosses are vertically ordered as follows: BYxRM, BYxYJM, BYxYPS, RMxYJM, RMxYPS, YJMxYPS. (B) The number of traits affected by loci within each 50-kb window. The grey dotted line shows the threshold for significance, while the black dotted line highlights the bins in which only one trait was affected.

For each trait, we expected to detect loci at the same genomic positions in different crosses sharing a parent. To identify only the distinct loci affecting each trait, we performed a grouping procedure on the peaks identified in all crosses for a given chemical condition. We found 411 distinct loci (an average of 32 loci per condition), with a minimum of 8 loci for growth in cycloheximide and a maximum of 57 loci for growth in zeocin (Figure 1 and Figure 2A). We then examined the extent to which these loci showed effects on growth in multiple conditions. For a range of genomic window sizes, we considered peaks detected for multiple chemicals within a window to correspond to the same underlying locus, and counted the number of conditions in which the locus showed an effect. With 50-kilobase (kb) windows, we found that 40% of the distinct loci had effects in only one condition, 29% had effects in two conditions, 11% had effects in three conditions, and only 20% had effects in four or more conditions (Figure 2B; Materials and Methods). Although the numbers differed across window sizes, the general observation that most of the detected loci had effects in a relatively small number of the tested conditions, and only a small number of loci showed effects across a large number of conditions, held over the entire range of plausible sizes. With 50 kb windows, three loci exhibited effects in more conditions than expected by chance (Materials and Methods). These loci were located on Chromosome V near the X-QTL control marker *CAN1*, Chromosome X near *ENT3*, *RSF2*, and *VPS70*, and Chromosome XIV near the pleiotropic gene *MKT1*.

We next examined the patterns of detection of loci for each trait across the six crosses. With four strains, two simple patterns are possible at bi-allelic loci: one strain can carry an allele that confers susceptibility or resistance relative to the allele carried by the other three strains, or two strains can carry the more susceptible allele and two strains the more resistant allele. We refer to these cases as “allelic singletons” and “allelic doubletons,” respectively. These two cases should give rise to different patterns of peaks: peaks with a consistent direction of effect in all three crosses involving one strain for allelic singletons, and peaks with specific effect directions in four specific crosses for allelic doubletons (Table S1; Table 1). Allowing for false-negative peaks, 135 of the 411 distinct loci showed patterns consistent with allelic singletons, and 28 showed patterns of peaks consistent with allelic doubletons (Table S1; Table 1).

**Table 1:** Patterns used to identify allelic singletons and allelic doubletons in the X-QTL data, and the number of loci detected with these patterns.

		Pattern of detection							
Allele	Effect	BYxRM	BYxYJM	BYxYPS	RMxYJM	RMxYPS	YJMxYPS	Subclass Total	Class Total
BY singleton	Resistant	BY	BY	BY	.	.	.	26	42
	Susceptible	RM	YJM	YPS	.	.	.	16	
RM singleton	Resistant	RM	.	.	RM	RM	.	19	49
	Susceptible	BY	.	.	YJM	YPS	.	30	
YJM singleton	Resistant	.	YJM	.	YJM	.	YJM	7	18
	Susceptible	.	BY	.	RM	.	YPS	11	
YPS singleton	Resistant	.	.	YPS	.	YPS	YPS	9	26
	Susceptible	.	.	BY	.	RM	YJM	17	
BY = RM doubleton	BY & RM resistant	.	BY	BY	RM	RM	.	7	12
	BY & RM susceptible	.	YJM	YPS	YJM	YPS	.	5	
BY = YJM doubleton	BY & YJM resistant	BY	.	BY	YJM	.	YJM	3	6
	BY & YJM susceptible	RM	.	YPS	RM	.	YPS	3	
BY = YPS doubleton	BY & YPS resistant	BY	BY	.	.	YPS	YPS	7	10
	BY & YPS susceptible	RM	YJM	.	.	RM	YJM	3	

The strain mentioned under a cross indicates which allele should have been selected in that cross for the given pattern to hold. Both exact match patterns and patterns that allow for one undetected peak were used to generate this table. A full listing of the exact match and “one off” patterns is described in Table S1.

We attempted to narrow the number of candidate genes for each of the bi-allelic loci by scanning the parental genome sequences for SNP alleles that are found in the four strains in a pattern consistent with the peaks. Using this approach, we found an average of 10 candidate genes per locus, with a range of 1 to 18 genes. Further restricting the list of candidate genes to those that carry nonsynonymous polymorphisms with appropriate allelic patterns reduced the average number to 6 per locus. We attempted to validate the genes underlying some of these loci by constructing allele replacement strains, and found reproducible evidence that *HXT6* and *RED1* harbor functional polymorphisms that confer growth differences in rich medium and tunicamycin, respectively (Figure S2; Materials and Methods). *HXT6* is a high affinity glucose transporter [21], suggesting that variability in glucose uptake may contribute to growth differences among the strains. The effect of *RED1* on tunicamycin resistance is less clear, as this gene is thought to be involved in chromosome segregation [21], and tunicamycin affects the unfolded protein response. We also constructed allele replacement strains for two other genes: *NUP157*, which lies within a copper sulfate resistance locus with the resistance allele coming from BY, and *PTK1*, which lies within a paraquat resistance locus with the resistance allele coming from YPS. However, we obtained inconsistent results for *NUP157* and *PTK1*: the allele replacements produced effects on resistance that were in the opposite direction from those seen in the X-QTL selections, and also caused growth defects on standard rich medium, suggesting that we did not identify the right candidate genes for these loci.

In addition to the simple bi-allelic patterns, we observed other more complex patterns of peaks (Figure 2A). Some of these are consistent with the presence of allelic series, in which either three or four alleles with different phenotypic effects are present among the four strains; we observed 29 examples involving at least 3 alleles and 9 examples that can only be explained by the presence of 4 different alleles (Table S2). The other 210 loci (51% of all loci) showed patterns of peaks that were not easily interpretable in terms of specific allelic classes. This probably reflects a mixture of false negatives in which a peak was present but not detected in a given cross, and cross-specific effects due to non-additive interactions and linkage between loci.

**Figure 3:**
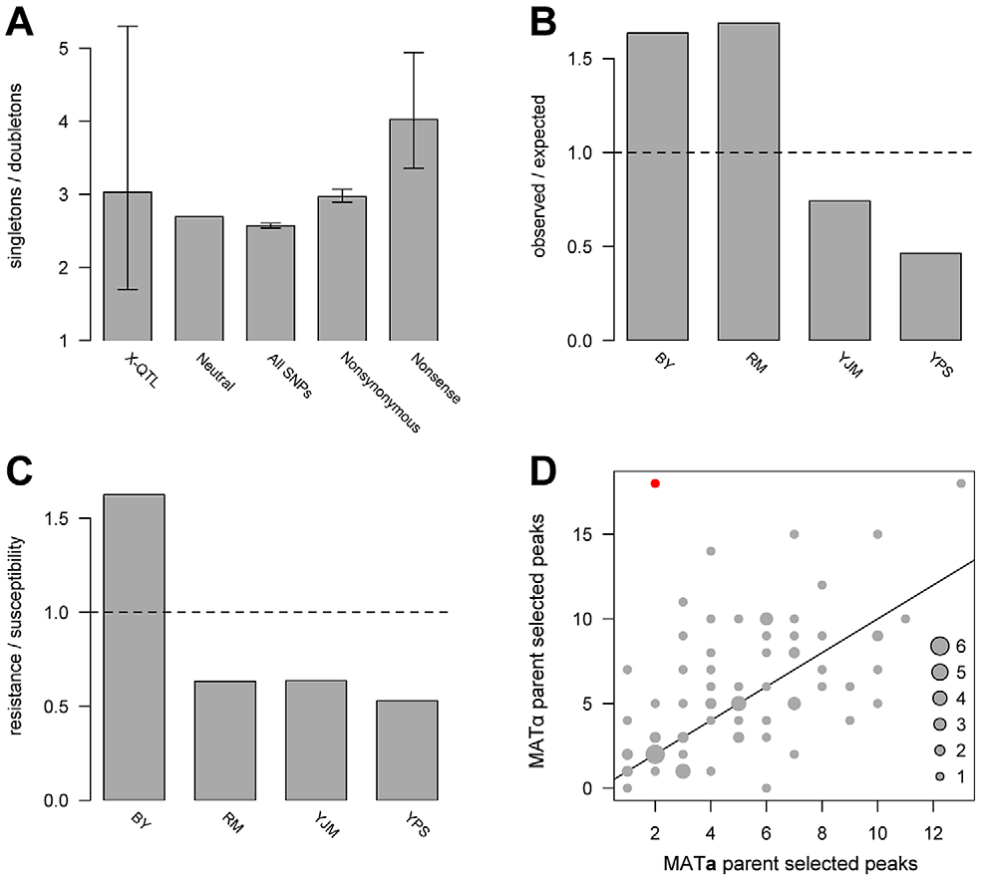
Population genetics of identified loci. (A) shows the ratios of singletons to doubletons observed in the X-QTL data and for different classes of sequence variation, (B) plots the ratio of observed X-QTL singletons to expected singletons by strain, (C) plots the ratio of resistance-conferring singletons to susceptibility-conferring singletons by strain, and (D) plots the directionalities of peaks detected in the 78 X-QTL selections. In A, the error bars denote 95% confidence intervals. For the maximum likelihood estimate of the ratio of singletons to doubletons among X-QTL loci, the confidence interval was determined from the likelihood surface. For classes of sites analyzed in the resequencing data, confidence intervals were obtained using bootstrapping. The neutral estimate (8/3) is derived from a folded allele frequency spectrum for n = 4. The other three measurements were obtained directly from a multiple sequence alignment of the genome sequences of the four strains. In B, the number of expected allelic singletons per strain was determined by multiplying the total number of allelic singletons detected by the proportion of all SNP allelic singletons among the four strains present in that parental genome. The values in C were obtained from Table 1. The horizontal lines in B and C indicate a ratio of one. In D, the number of peaks selected in each direction in each X-QTL selection is plotted. One experiment—copper sulfate in the BYxRM cross—was significant for the sign test after a Bonferroni correction for multiple testing, indicating putative directional selection, and is shown in red. The number of experiments observed with a given number of up and down peaks is indicated by the size of a circle, with a key provided in the bottom right corner. The diagonal line in D shows a 1∶1 ratio of peaks selected in the direction of each parent.

The allele frequency spectrum of causal loci is critical for the design of genetic mapping studies and for understanding sources of missing heritability in natural populations, including humans. As discussed above, we were able to distinguish and enumerate two simple allelic classes—singletons and doubletons. We used a maximum likelihood approach that accounted for false negatives to estimate the ratio of allelic singletons to doubletons. We estimated the peak detection rate to be 51%, with a 95% confidence interval of 39%–62%, and the ratio of allelic singletons to doubletons to be 3.03, with a 95% confidence interval of 1.7–5.3 (Figure 3A; Figure S3). This result suggests that despite the high statistical power of X-QTL, a substantial fraction of loci with weaker effects likely still go undetected in any one cross. Interestingly, the estimate of the ratio of allelic singletons to doubletons is similar to that observed for nonsynonymous polymorphisms in the genomes of the parent strains (2.97), and is shifted toward singletons relative to both the neutral expectation of 2.67 and the observed ratio of 2.57 for 109,585 SNPs genome-wide (Figure 3A). Thus, the frequency spectrum of variants that contribute to complex trait variation in yeast appears to be mildly shifted toward lower frequencies by purifying selection, but, given the wide confidence interval for the estimated ratio of allelic singletons to doubletons, we cannot rule out that the variant frequencies follow the neutral spectrum.

Several lines of evidence suggest that lineage-specific selection or demography has shaped variation among the four strains. We observed an excess of allelic singletons at detected loci for BY and RM, and a deficit for YJM and YPS, relative to the numbers of singleton SNPs in the parent genomes (χ2 = 35.98; d.f. = 3, p<0.0001; Figure 3B). The laboratory strain BY also exhibits other signatures of selection for both general and chemical-specific resistance. For instance, BY carries a marginally significant excess of allelic singletons that confer resistance relative to the other three strains (Fisher's exact test, Bonferroni-corrected p = 0.06; Figure 3C; Table 1). In addition, trait-specific sign tests [22] identified one significant result: an excess of copper sulfate resistance alleles contributed by BY in the BYxRM cross (18 loci with BY carrying the resistance allele and 2 loci with RM carrying the resistance allele; binomial test, Bonferroni-corrected p = 0.031; Figure 3D). Interestingly, BY is among the most copper-resistant *S. cerevisiae* strains [23], [24], and our data suggest that this resistance in BY may be the result of selection, possibly due to the use of high levels of copper or another chemical with similar effects in standard growth media. However, the BYxYJM and BYxYPS crosses do not show significant excess of BY alleles, and RM is also among the more highly copper-resistant strains [23], making the excess of BY resistance alleles in the BYxRM cross difficult to explain. Overall, our results are consistent with previous analyses that have shown lab strains isogenic to BY exhibit high evolutionary rates relative to other yeast isolates [25], probably due to both relaxed purifying selection [26] and adaptation [26], [27].

We have shown that variation in chemical resistance among yeast strains is typically due to a large number of underlying loci. The level of genetic complexity, as measured by the number of loci detected, is largely a property of each resistance trait, although it is also affected to a lesser extent by the choice of parent strains. The total number of distinct loci detected for a trait in these crosses among four strains ranged from 8 to 57, and these numbers substantially exceeded those seen in any one cross. These observations suggest that the total number of loci affecting certain resistance traits in *S. cerevisiae* can be very large, since many of them will have escaped detection because they don't vary among the four parent strains examined here, have effect sizes that are too small, or are too closely linked to be resolved as separate loci by our mapping technique. Our results suggest that the functional variants underlying complex traits are broadly distributed across the frequency spectrum from rare to common alleles, and that many loci harbor more than two allelic variants. These findings provide multiple non-exclusive explanations for the sources of the “missing heritability” of complex traits, and illustrate the power of a simple model system for probing genetic complexity.

## Materials and Methods

### Construction and use of segregating pools for X-QTL

The Synthetic Genetic Array marker system [28] was used to generate MAT**a** haploid pools as previously reported [5], with the exception that thialysine and the dominant sensitive *LYP1*/*lyp1Δ* marker system were not employed. All six pairwise crosses of BY, RM, YJM, and YPS were made, with one strain in a cross having the genotype MATα *can1Δ::STE2pr-SpHIS5 his3Δ* and the other having the genotype MAT**a** *his3Δ*. In notation describing crosses (e.g., BYxRM), we first list the MATα and then the MAT**a** parent. The selection experiments used for X-QTL were conducted as previously described [5]. The drug doses used in the selections, which were determined by plating millions of cells across a range of drug doses and finding a concentration at which 300 to 1,000 colonies could be resolved, are given in File S1. Each experiment was conducted once, as we previously found that biological replicates conducted on the same day produced highly similar results [5].

### Microarray design and use

Microarrays were designed from the BY genome sequence obtained from the *Saccharomyces* Genome Database (http://www.yeastgenome.org/) and from assemblies of the RM, YJM, and YPS genomes obtained from the *Saccharomyces* Genome Resequencing Project [10]. Note that the YPS606 genome was used to design the YPS array, as YPS606 is isogenic to YPS163. We aligned the genomes chromosome-by-chromosome using Fast Statistical Alignment (FSA) [29]. These multiple sequence alignments were filtered for SNPs using the following criteria: i) all 4 strains had to have been sequenced at a position and ii) all 4 strains had to have a specific base called (i.e. A, C, G, or T) at the position. These SNPs were then used for microarray design, as well as for downstream population-genetic analyses. Cross-specific microarrays were designed using only bi-allelic SNPs. Probes were chosen to have a length between 21 and 27 nucleotides and a melting temperature between 54 and 56°C as described previously [5], [30]. One probe was designed for each allele of a SNP, and the two probes for a SNP were randomly positioned on the microarray. Probes were targeted to regions where only one SNP would be covered by the probes. Markers were chosen to provide near-uniform coverage of the genome. The arrays were tested using control DNA from both parents and the heterozygous diploid to ensure that they could discriminate the two alleles of a SNP. All hybridizations and processing was done as previously described [5]. All microarray data is available in the Princeton University MicroArray database (http://puma.princeton.edu/). The processed log_10_ hybridization intensities are included in Files S2, S3, S4, S5, S6, S7.

### Peak detection

For a given SNP, the difference in the log_10_ ratios of the intensities of the MATα and MAT**a** parent-specific probes on a single array was computed (subsequently referred to as a ‘log_10_ intensity difference’), and this metric was used in downstream analyses. Background allele frequency changes that occur during pool construction were removed from the data for each X-QTL selection. This was done separately for each SNP by subtracting the average log_10_ intensity difference obtained in seven cross-specific control experiments from the log_10_ intensity difference observed in an X-QTL selection. A peak detection algorithm was then employed that used a Savitzky-Golay filter to smooth the data within sliding windows of 100 probes. This smoothing approach was used to preserve local maxima in the data. Loci were called at a 1% FDR threshold, where the number of false discoveries was determined by running the peak caller on the control data using a range of thresholds, and the total number of discoveries was determined by running the peak caller on the selection data at the same thresholds used to analyze the controls. Thresholds were set by examining the quantiles of log_10_ intensity differences observed for every 100 SNP genomic window on an array, and taking the median interquantile range between the x and 1-x quantiles, where x ranged from 0.005 to 0.45. We found that setting x as 0.045 resulted in a 1% FDR. Peak calling and all other statistical analyses were conducted in R (http://www.r-project.org/). The peak caller and an associated function library are included in Files S8 and S9. The detected peaks are listed in File S10.

### Testing for effects of chemical and genetic background on the number of peaks detected in a cross

The test for cross effect was conducted using the model y = chemical+cross, while the test for strain effect was conducted using the model y = chemical+strain1+strain2+strain3. Implementing the second test required specifying the design matrix for the strain effect. Each row in the design matrix represented a single X-QTL experiment from a particular combination of chemical and cross. Entries in the design matrix were parameterized as follows: a strain had a value of −1, 1, or 0 if it was the MAT**a** parent, the MATα parent, or not a parent in a particular experiment, respectively. Only three strains were included in the test, because the information for the fourth could be obtained from the other three. To ensure that results were not dependent on the three included strains, we conducted the test with all four possible combinations of the three strains and reported the maximum partial R^2^ and F values, and the minimum p value in the text.

### Testing for disproportionate contributions of particular strains to the genetic complexity of traits

We first conducted χ2 tests in which single strains were examined. This test has two categories – one that is the sum of the peaks detected in the three crosses involving the query strain and another that is the sum of the peaks detected in the other three crosses. The expectation is that each of these classes will contain half of the peaks detected for a trait. We then conducted χ2 tests in which two strains were examined. The first category here is the sum of the peaks detected in the four crosses involving the two strains, while the second is sum of the peaks detected in the other two crosses. Here, the expectation is that the first category will contain two-thirds of the peaks, while the second will contain one-third of the peaks.

### Identification of distinct loci for a trait

Peaks identified across the six crosses for a single trait were grouped into distinct loci. We started with the most strongly selected peak on each chromosome and grouped with it all peaks that occurred within a 200-kilobase window surrounding it. This window size accommodated the grouping of peaks that exhibited weak but significant allele frequency changes, and may result in the underestimation of the total number of loci due to the overgrouping of peaks. Remaining peaks were grouped into distinct loci using additional iterations of the procedure until all peaks identified for a trait were members of a group.

### Analysis of distinct loci across traits

We divided the genome into equally sized bins ranging from 20 to 100 kb and counted the number of distinct loci that fell into each bin. A bin was considered to have an excess of distinct loci if the number present in it exceeded the number expected by chance from a Poisson distribution, given the number of distinct loci divided by the total number of bins and a multiple testing correction for the number of bins. With the 50 kb bin size reported in the text, 8 or more distinct loci were required to be present in a bin for the bin to be considered significant.

### Identification of allelic singletons, doubletons, and series

The distinct loci identified for each trait were used to classify singletons and doubletons. The specific patterns used to identify the allelic classes are described in Table S1. We focused on exact pattern matches and on patterns that were missing an expected peak at a given locus in one cross. A number of distinct loci had peaks detected in four or more crosses, but did not conform to the patterns expected for allelic doubletons. We considered these loci as allelic series, and for each of these putative series we determined the possible logical relationships of the parent alleles to each other. These relationships are reported in Table S2.

### Identification of candidate causal genes

For each bi-allelic locus, we evaluated a 30 kb interval centered on its estimated position for polymorphisms that segregated among the parent strains in the same pattern as the X-QTL peaks. Any gene that harbored a polymorphism in the coding region or in the immediate upstream and downstream regions was considered a candidate. The candidate genes are listed in File S11.

### Allele replacement strategy

To generate the replacement strains, we used the allele replacement technique described by Storici et al. [31]. This method is a two-step process that involves knocking out a gene with a selectable marker cassette, and then replacing the selectable marker cassette with a different allele of the gene. We made each allele replacement strain once in one parental background, and then compared the phenotypes of the strains to their progenitors. For the two genes that exhibited the expected phenotypic effect, we made a second version of the allele replacement strain to validate the presence of functional variation in the gene.

### Maximum likelihood estimation of the ratio of singletons to doubletons

The observed counts of exact-match allelic singletons and doubletons and near-exact-match allelic singletons and doubletons were modelled using two parameters: the detection rate of peaks (α) and the ratio of singletons to doubletons (β). The formulae underlying this computation are provided in Text S1. The likelihood of each combination of parameter values was examined across a two-dimensional grid of parameter values using χ2 tests with 3 degrees of freedom. The likelihood reached a maximum at α = 0.51 and β = 3.03. We obtained 95% confidence intervals for α and β by using the χ2 distribution with 3 degrees of freedom and identifying the χ2 value for the 95% quantile. We then identified parameter combinations that produced an χ2 value below this threshold (7.81), and determined the minimum and maximum values of α and β that satisfied this condition.

## Supporting Information

Figure S1 Plots of X-QTL mapping results. The data for each trait is plotted as the difference between the MATα and MATa allele-specific probes on the selection array minus the average of the differences between the MATα and MATa allele-specific probes from seven control arrays. The red vertical lines indicate positions that were called as peaks at a 1% FDR.
(DOC)

Figure S2 Cloning of genes. (A–C) show the steps taken to clone *HXT6*, while (D–E) show the steps taken to clone *RED1*. In both cases, a locus was identified in all three crosses sharing one parent—the crosses involving RM in control conditions for *HXT6* (A) and the crosses involving BY on tunicamycin for *RED1* (B). The regions underlying the detected peaks were surveyed for polymorphisms that segregated across the parent strains in the same pattern as the detected locus. Both *HXT6* and *RED1* were chosen because they carry a number of nonsynonymous polymorphisms relative to other genes in their genomic regions (B and E). Allele replacement strains were made in the RM background using the BY strain as a template. Each strain was independently constructed twice and phenotyped using serially diluted colony growth assays (C and F). Overnight cultures were grown for each strain and then pinned onto agar plates using the Singer RoToR. The *HXT6* strains were measured after 24 hours of growth at 30°C, while the *RED* strains were measured after 65 hours of growth at 30°C. RM grows better on standard medium when it carries its own allele of *HXT6*, while the BY allele of *RED1* confers a growth advantage on tunicamycin. In B, dubious ORFs are colored in blue. In C and F, cultures were grown undiluted (abbreviated “Und.”) and at two successive ten-fold dilutions. Each dilution of a strain was pinned in a square of four technical replicates.
(DOC)

Figure S3 Likelihood surface for the estimates of the ratio of allelic singletons to doubletons and the detection rate. P(Data|Model) is shown, with the correspondence of colors to probabilities given in the key. This was generated using the model described in Text S1, and evaluating the model across a wide, two-dimensional range of detection rates and singleton to doubleton ratios. In addition, this likelihood surface was used to generate the confidence intervals described in the main text.
(DOC)

File S1 Results from dose-response experiments with segregant pools and final drug doses used in the paper.
(XLSX)

File S2 Processed log_10_ hybridization intensities for the BYxRM cross.
(TXT)

File S3 Processed log_10_ hybridization intensities for the BYxYJM cross.
(TXT)

File S4 Processed log_10_ hybridization intensities for the BYxYPS cross.
(TXT)

File S5 Processed log_10_ hybridization intensities for the RMxYJM cross.
(TXT)

File S6 Processed log_10_ hybridization intensities for the RMxYPS cross.
(TXT)

File S7 Processed log_10_ hybridization intensities for the YJMxYPS cross.
(TXT)

File S8 Peak caller.
(R) Click here for additional data file.

File S9 Library for the peak caller.
(R) Click here for additional data file.

File S10 Loci detected in the X-QTL experiments.
(TXT)

File S11 Candidate genes for bi-allelic loci.
(TXT)

Table S1 All patterns used to identify allelic singletons and allelic doubletons in the X-QTL data, and the number of loci detected with these patterns. The strain mentioned under a cross indicates which allele should have been selected in that cross for the given pattern to hold. We show all exact patterns used to identify singletons and doubletons, as well as each of the patterns that indicate the presence of a singleton or doubleton if one undetected peak is allowed. Both the exact match and “one off” patterns were used in the counts of bi-allelic loci described throughout the paper.
(DOC)

Table S2 Allelic series inferred from the data. The most parsimonious relationship of alleles to each other is indicated. Greater than and equal signs indicate the effects of the alleles relative to each other, with “A>B” meaning that allele A confers higher resistance than allele B and “A = B” meaning that the effects of allele A and allele B are not distinguishable. In some cases, there are two equally parsimonious relationships that can explain the data.
(DOC)

Text S1 Formulae used to estimate the detection rate (α) and the ratio of allelic singletons to doubletons (β).
(DOC)
