# Supplementary material for: Genetic Architecture of Highly Complex Chemical Resistance Traits across Four Yeast Strains
Source: PLoS Genet. 2012 Mar 15;8(3):e1002570. doi: 10.1371/journal.pgen.1002570 (PMC3305394; doi:10.1371/journal.pgen.1002570)
Supplement: Figure S1 — Plots of X-QTL mapping results. The data for each trait is plotted as the difference between the MATα and MATa allele-specific probes on the selection array minus the average of the differences between the MATα and MATa allele-specific probes from seven control arrays. The red vertical lines indicate positions that were called as peaks at a 1% FDR. (DOC) [file pgen.1002570.s001.doc]

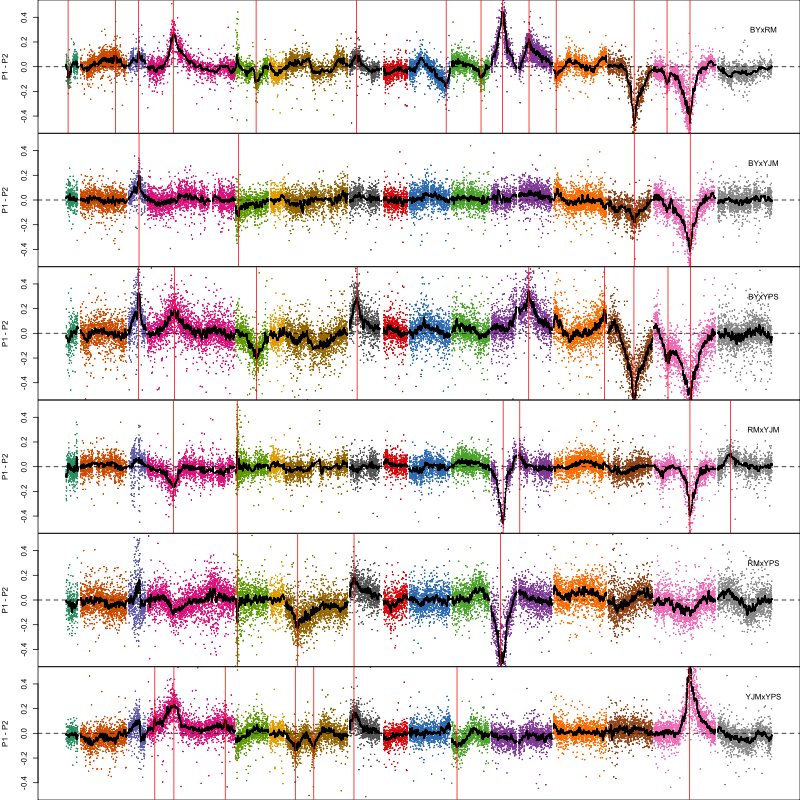


**Supplementary Figure 1A. Loci detected for 4-NQO.**

**
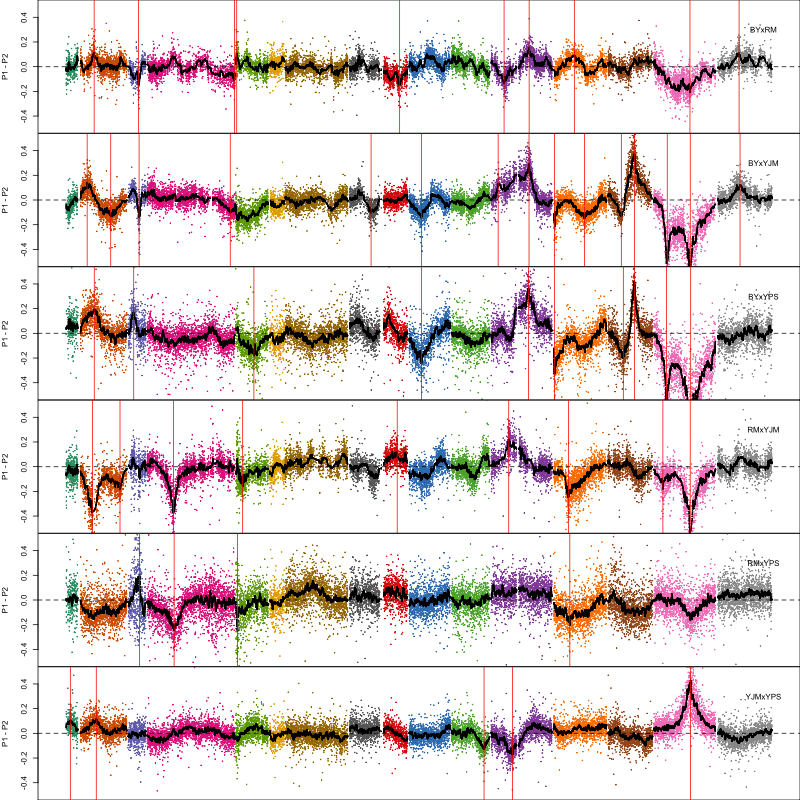
**

**Supplementary Figure 1B. Loci detected for Benomyl.**

**
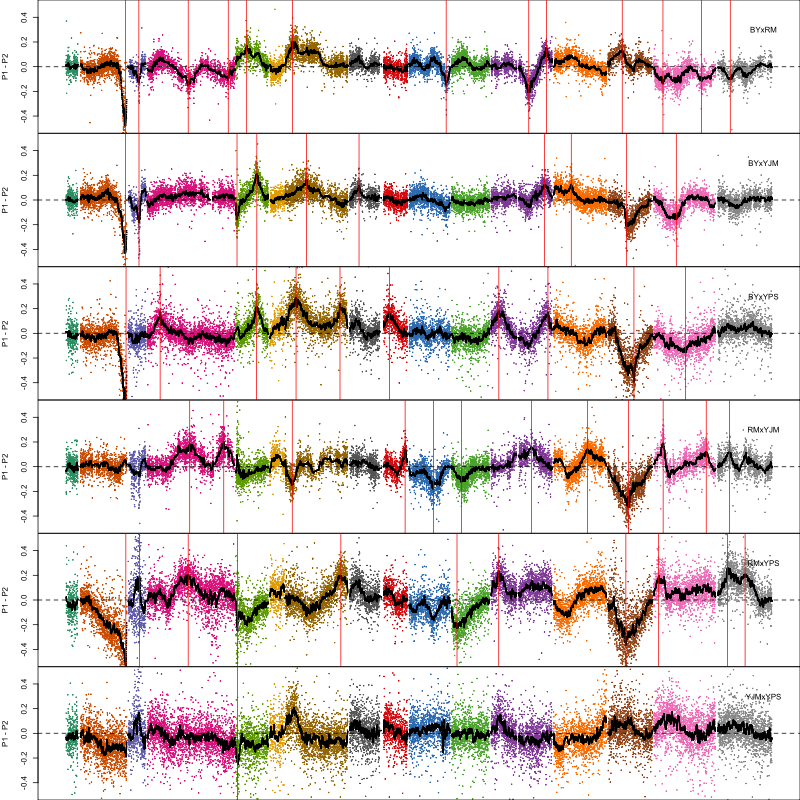
**

**Supplementary Figure 1C. Loci detected for cadmium chloride.**

**
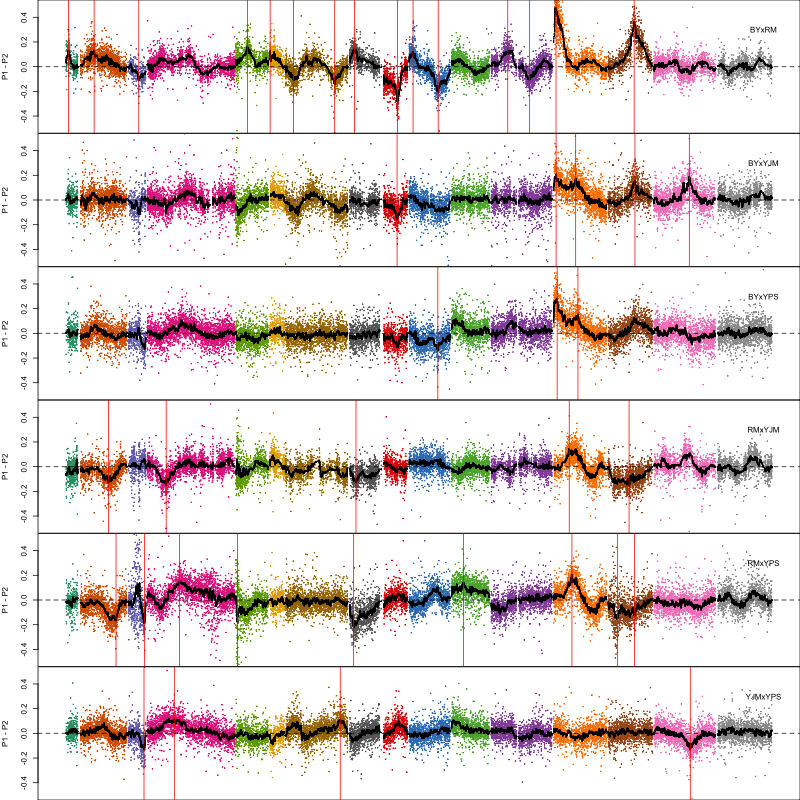
**

**Supplementary Figure 1D. Loci detected for chlorpromazine.**

**
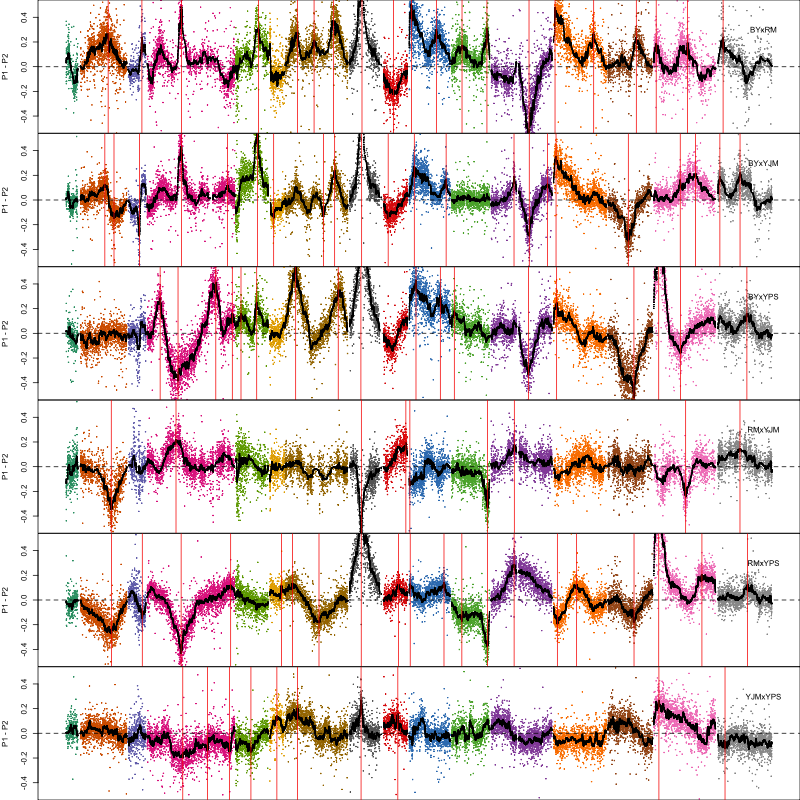
**

**Supplementary Figure 1E. Loci detected for copper sulfate.**

**
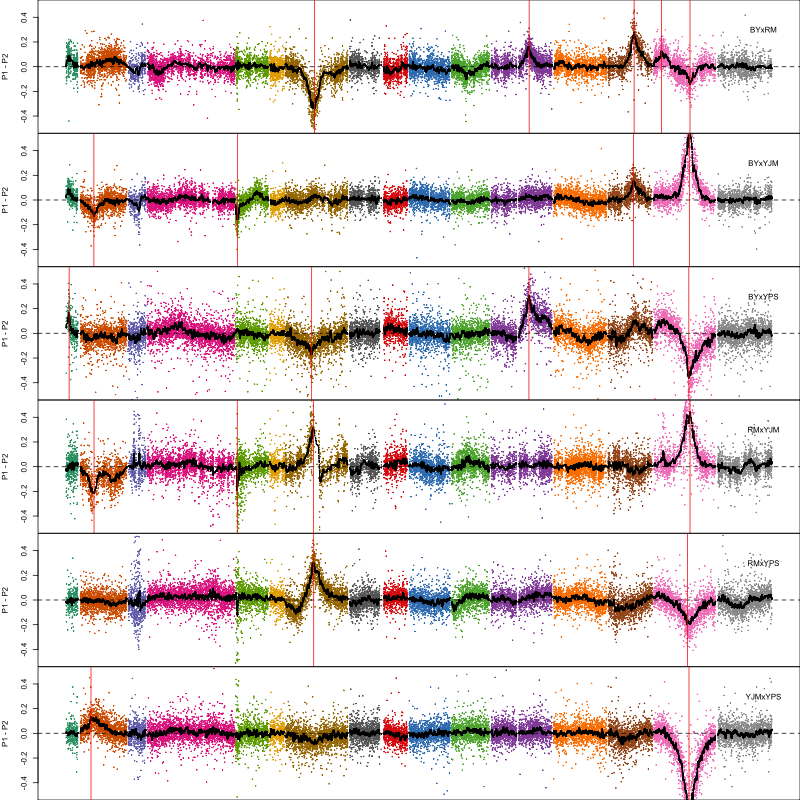
**

**Supplementary Figure 1F. Loci detected for cycloheximide.**

**
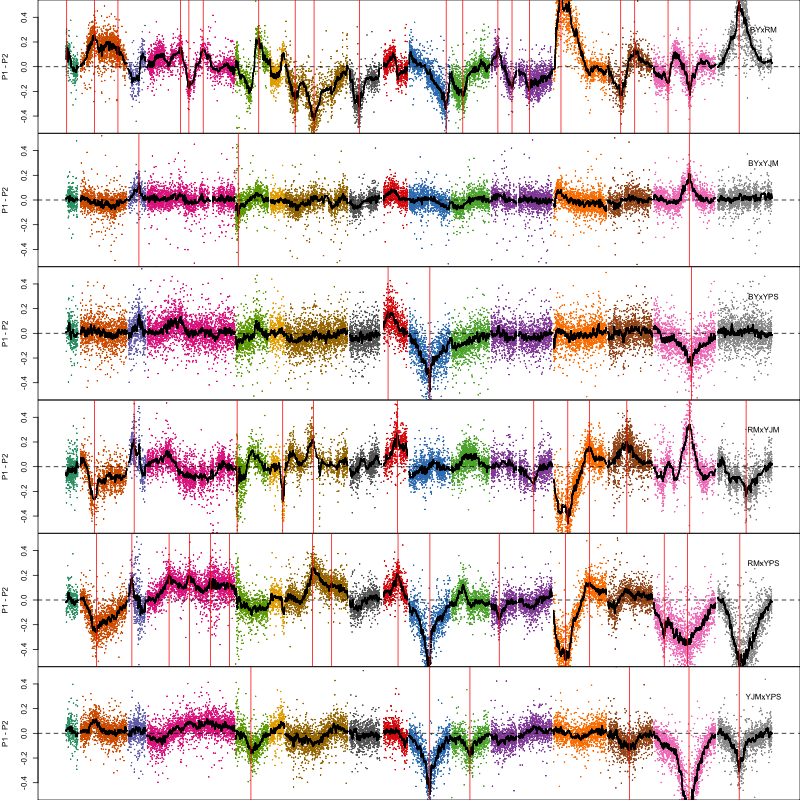
**

**Supplementary Figure 1G. Loci detected for diamide.**

**
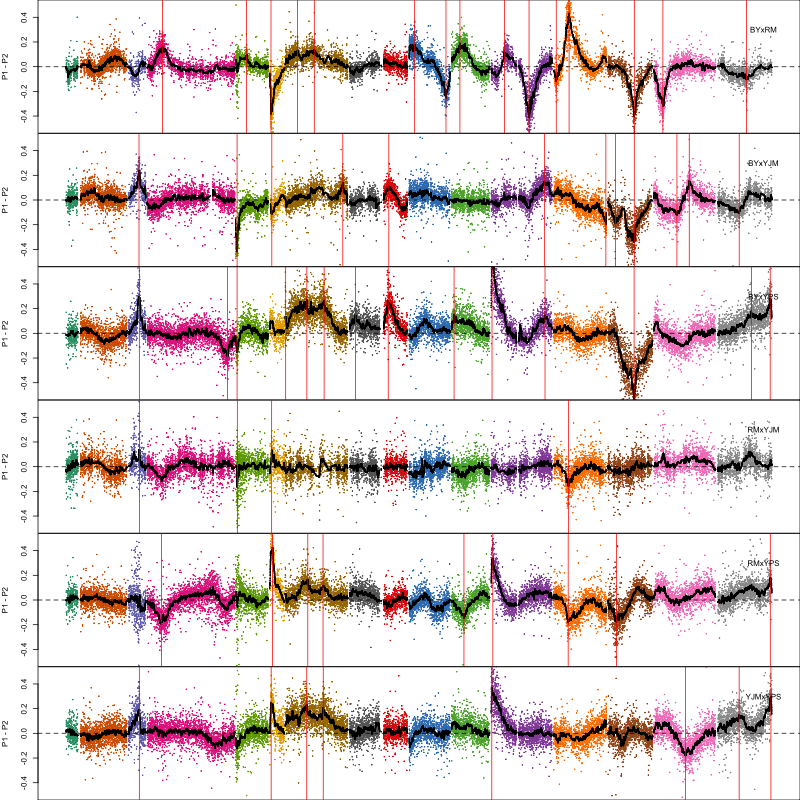
**

**Supplementary Figure 1H. Loci detected for hydrogen peroxide.**

**
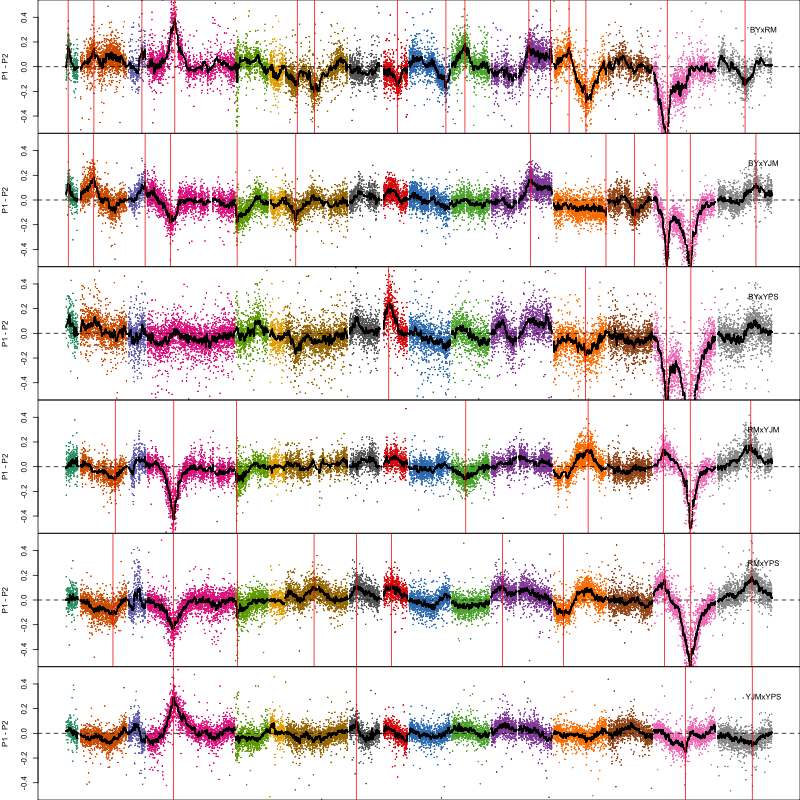
**

**Supplementary Figure 1I. Loci detected for LY83583.**

**
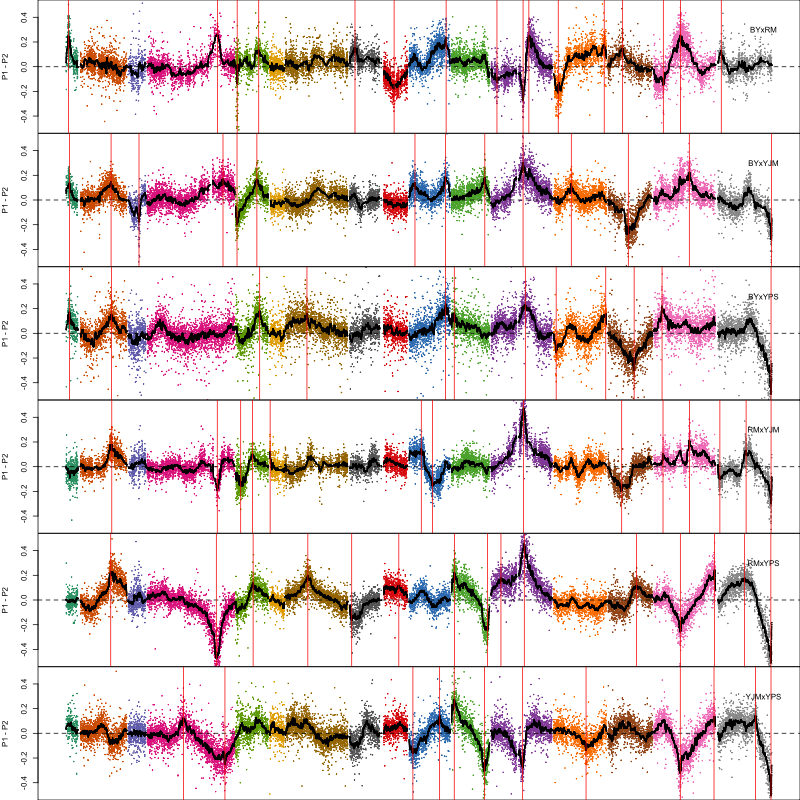
**

**Supplementary Figure 1J. Loci detected for paraquat.**

**
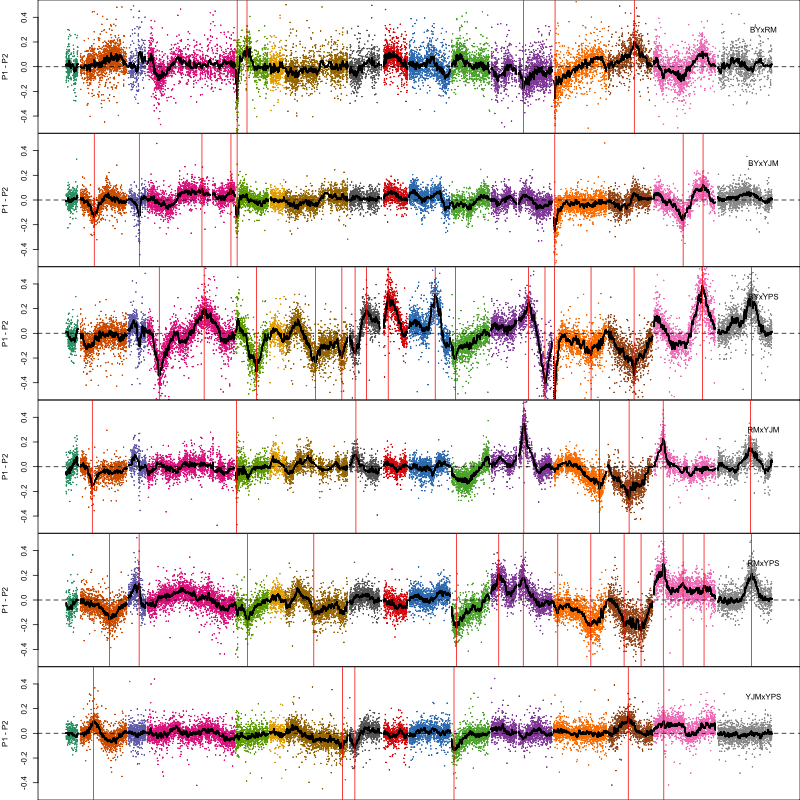
**

**Supplementary Figure 1K. Loci detected for SDS.**

**
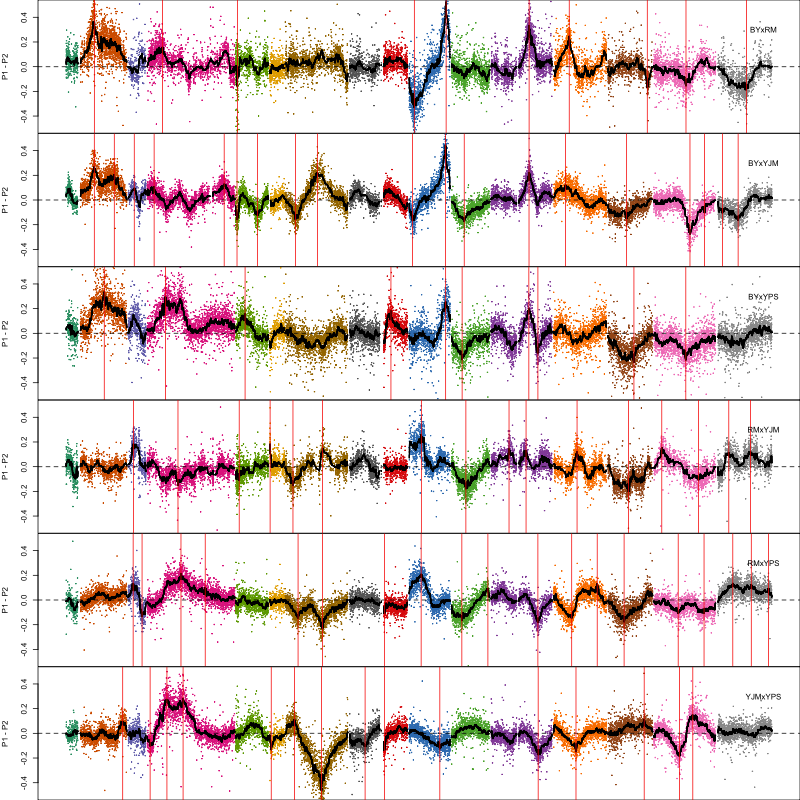
**

**Supplementary Figure 1L. Loci detected for tunicamycin.**

**
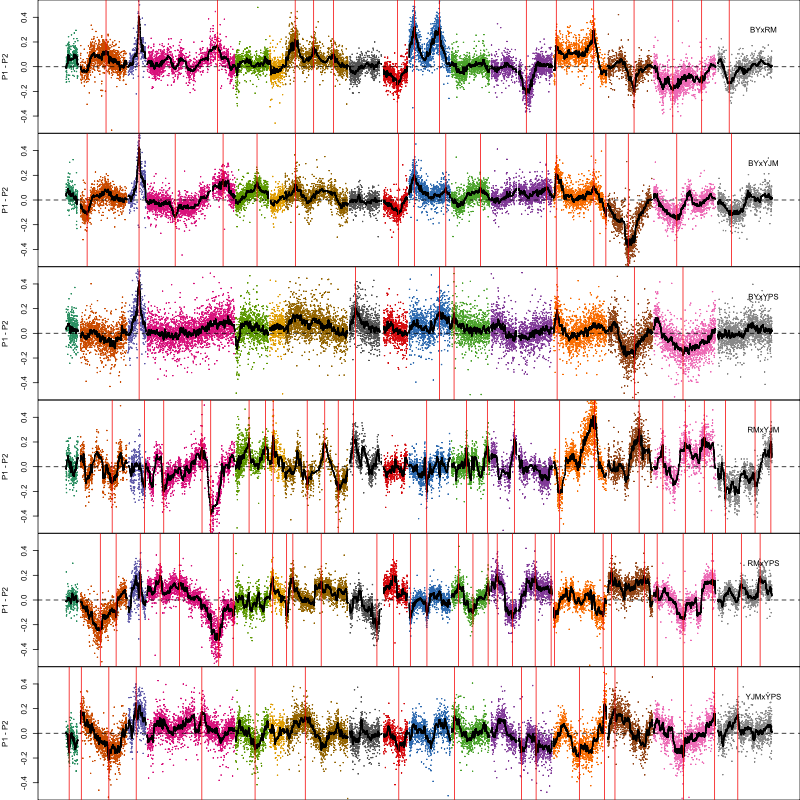
**

**Supplementary Figure 1M. Loci detected for zeocin.**
