# Supplementary figures and images for: Genetic Architecture of Highly Complex Chemical Resistance Traits across Four Yeast Strains
Source: PLoS Genet. 2012 Mar 15;8(3):e1002570. doi: 10.1371/journal.pgen.1002570 (PMC3305394; doi:10.1371/journal.pgen.1002570)

**
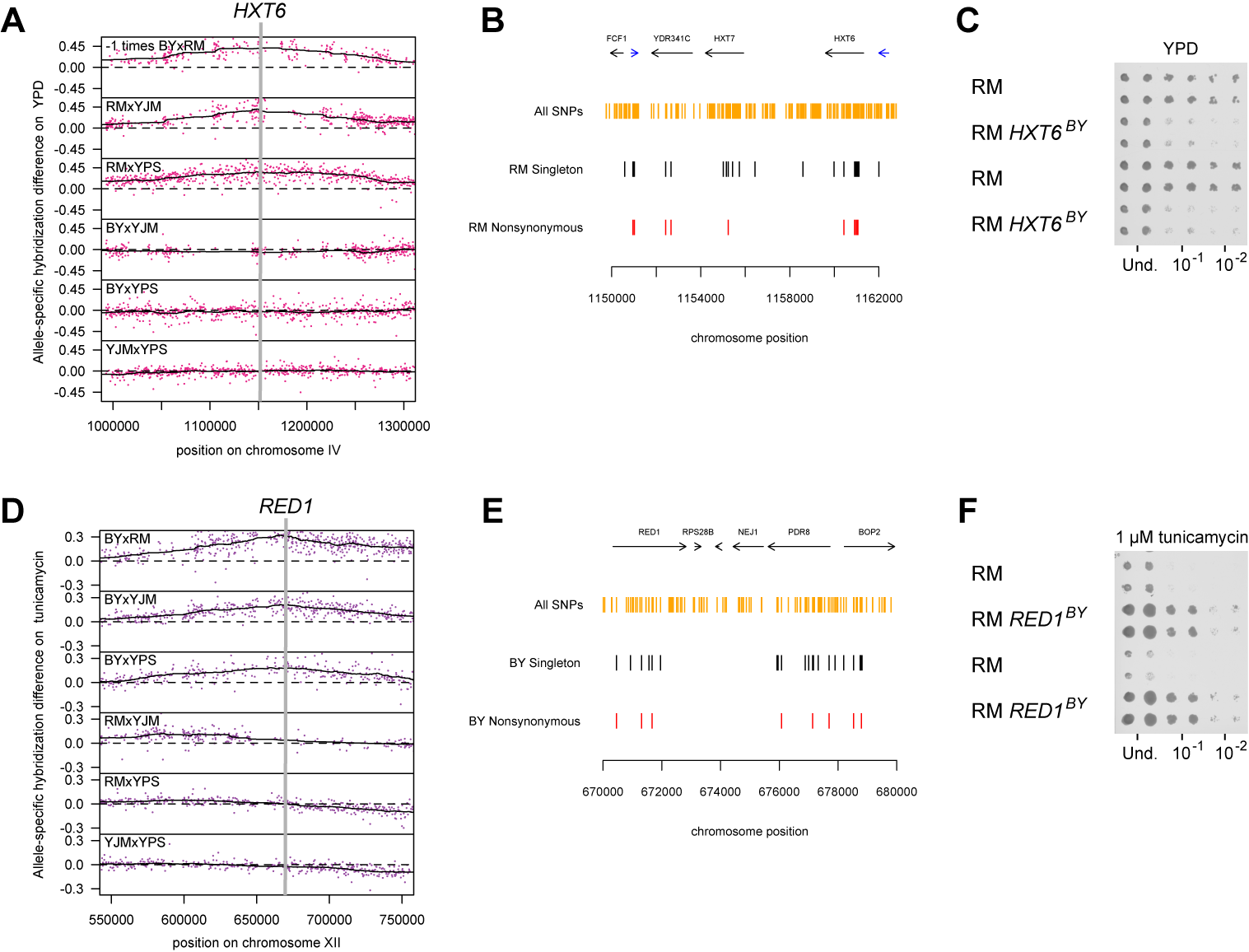
**

Supplement: Figure S2 — Cloning of genes. (A–C) show the steps taken to clone HXT6, while (D–E) show the steps taken to clone RED1. In both cases, a locus was identified in all three crosses sharing one parent—the crosses involving RM in control conditions for HXT6 (A) and the crosses involving BY on tunicamycin for RED1 (B). The regions underlying the detected peaks were surveyed for polymorphisms that segregated across the parent strains in the same pattern as the detected locus. Both HXT6 and RED1 were chosen because they carry a number of nonsynonymous polymorphisms relative to other genes in their genomic regions (B and E). Allele replacement strains were made in the RM background using the BY strain as a template. Each strain was independently constructed twice and phenotyped using serially diluted colony growth assays (C and F). Overnight cultures were grown for each strain and then pinned onto agar plates using the Singer RoToR. The HXT6 strains were measured after 24 hours of growth at 30°C, while the RED strains were measured after 65 hours of growth at 30°C. RM grows better on standard medium when it carries its own allele of HXT6, while the BY allele of RED1 confers a growth advantage on tunicamycin. In B, dubious ORFs are colored in blue. In C and F, cultures were grown undiluted (abbreviated “Und.”) and at two successive ten-fold dilutions. Each dilution of a strain was pinned in a square of four technical replicates. (DOC) [file pgen.1002570.s002.doc]

**
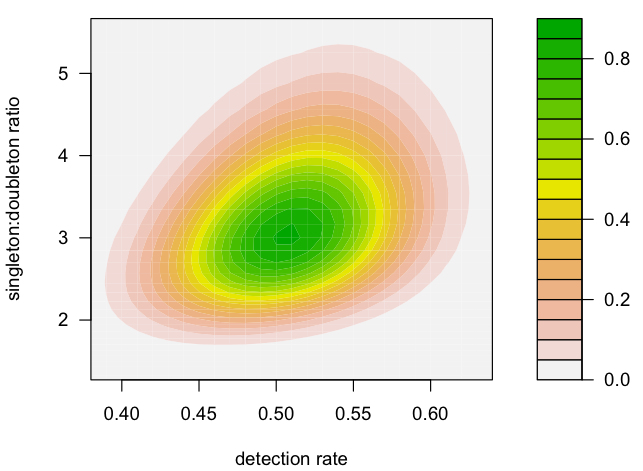
**

Supplement: Figure S3 — Likelihood surface for the estimates of the ratio of allelic singletons to doubletons and the detection rate. P(Data|Model) is shown, with the correspondence of colors to probabilities given in the key. This was generated using the model described in Text S1, and evaluating the model across a wide, two-dimensional range of detection rates and singleton to doubleton ratios. In addition, this likelihood surface was used to generate the confidence intervals described in the main text. (DOC) [file pgen.1002570.s003.doc]
