# Supplementary material for: Genetic Architecture of Highly Complex Chemical Resistance Traits across Four Yeast Strains
Source: PLoS Genet. 2012 Mar 15;8(3):e1002570. doi: 10.1371/journal.pgen.1002570 (PMC3305394; doi:10.1371/journal.pgen.1002570)
Supplement: Text S1 — Formulae used to estimate the detection rate (α) and the ratio of allelic singletons to doubletons (β). (DOC) [file pgen.1002570.s017.doc]

**Supplementary Note 1. Formulae used to estimate the detection rate (α) and the ratio of allelic singletons to doubletons (β).** We modelled four quantities in the data as a function of these two parameters—the number of allelic singletons that exactly matched the expected pattern (S_Exact_), the number of allelic doubletons that exactly matched the expected pattern (D_Exact_), the number of allelic singletons where one of three required crosses was not detected (S_Inexact_), and the number of allelic doubletons where one of four required crosses was not detected (D_Inexact_).

S_Exact_ = (163 × (β/(1 + β) × α^3^)/((β /(1 + β) × α^3^) + (β/(1 + β) × 3 × (α^2^× (1 - α))) + ((1- β/(1 + β)) × α^4^) + ((1 - β/(1 + β)) × 4 × (α^3^ × (1- α)))))

S_Inexact_ = (163 × (β/(1 + β) × 3 × (α^2^ × (1 - α)))/((β /(1 + β) × α^3^) + (β/(1 + β) × 3 × (α^2^× (1 - α))) + ((1- β/(1 + β)) × α^4^) + ((1 - β/(1 + β)) × 4 × (α^3^ × (1- α)))))

D_Exact_ = (163 × ((1- β/(1 + β)) × α^4^)/ ((β /(1 + β) × α^3^) + (β/(1 + β) × 3 × (α^2^× (1 - α))) + ((1- β/(1 + β)) × α^4^) + ((1 - β/(1 + β)) × 4 × (α^3^ × (1- α)))))

D_Inexact_ = (163 × ((1 - β/(1 + β)) × 4 × (α^3^ × (1- α)))/((β /(1 + β) × α^3^) + (β/(1 + β) × 3 × (α^2^× (1 - α))) + ((1- β/(1 + β)) × α^4^) + ((1 - β/(1 + β)) × 4 × (α^3^ × (1- α)))))

163 is the total number of bi-allelic loci detected by X-QTL. We calculated the fit of the model to the data using a χ^2^ statistic.

χ^2^ = (S_ExactData_ - S_ExactModel_)2/S_ExactModel_ + (D_ExactData_ - D_ExactModel_)2/D_ExactModel_ + (S_InexactData_ - S_InexactModel_)2/S_InexactModel_ + (D_InexactData_ - D_InexactModel_)2/D_InxactModel_

Where S_ExactData_ equals 36, S_InexactData_ equals 99, D_ExactData_ equals 4, and D_InexactData_ equals 24.
