# Supplementary material for: Genetic Architecture of Highly Complex Chemical Resistance Traits across Four Yeast Strains
Source: PLoS Genet. 2012 Mar 15;8(3):e1002570. doi: 10.1371/journal.pgen.1002570 (PMC3305394; doi:10.1371/journal.pgen.1002570)
Supplement: Supplementary file 15 [file pgen.1002570.s015.doc]

		Pattern of detection							Subclass	Class
Allele	Effect	BYxRM	BYxYJM	BYxYPS	RMxYJM	RMxYPS	YJMxYPS	Counts	Total	Total
BY singleton	Resistant	BY	BY	BY	.	.	.	10	26	42
		.	BY	BY	.	.	.	5		
		BY	.	BY	.	.	.	4		
		BY	BY	.	.	.	.	7		
	Susceptible	RM	YJM	YPS	.	.	.	3	16	
		.	YJM	YPS	.	.	.	3		
		RM	.	YPS	.	.	.	4		
		RM	YJM	.	.	.	.	6		
RM singleton	Resistant	RM	.	.	RM	RM	.	5	19	49
		.	.	.	RM	RM	.	5		
		RM	.	.	.	RM	.	4		
		RM	.	.	RM	.	.	5		
	Susceptible	BY	.	.	YJM	YPS	.	7	30	
		.	.	.	YJM	YPS	.	9		
		BY	.	.	.	YPS	.	10		
		BY	.	.	YJM	.	.	4		
YJM singleton	Resistant	.	YJM	.	YJM	.	YJM	2	7	18
		.	.	.	YJM	.	YJM	1		
		.	YJM	.	.	.	YJM	2		
		.	YJM	.	YJM	.	.	2		
	Susceptible	.	BY	.	RM	.	YPS	0	11	
		.	.	.	RM	.	YPS	3		
		.	BY	.	.	.	YPS	6		
		.	BY	.	RM	.	.	2		
YPS singleton	Resistant	.	.	YPS	.	YPS	YPS	3	9	26
		.	.	.	.	YPS	YPS	3		
		.	.	YPS	.	.	YPS	1		
		.	.	YPS	.	YPS	.	2		
	Susceptible	.	.	BY	.	RM	YJM	6	17	
		.	.	.	.	RM	YJM	6		
		.	.	BY	.	.	YJM	0		
		.	.	BY	.	RM	.	5		
BY = RM doubleton	BY & RM Resistant	.	BY	BY	RM	RM	.	1	7	12
		.	.	BY	RM	RM	.	1		
		.	BY	.	RM	RM	.	4		
		.	BY	BY	.	RM	.	1		
		.	BY	BY	RM	.	.	0		
	YJM & YPS Resistant	.	YJM	YPS	YJM	YPS	.	1	5	
		.	.	YPS	YJM	YPS	.	0		
		.	YJM	.	YJM	YPS	.	2		
		.	YJM	YPS	.	YPS	.	0		
		.	YJM	YPS	YJM	.	.	2		
BY = YJM doubleton	BY & YJM Resistant	BY	.	BY	YJM	.	YJM	1	3	6
		.	.	BY	YJM	.	YJM	0		
		BY	.	.	YJM	.	YJM	0		
		BY	.	BY	.	.	YJM	1		
		BY	.	BY	YJM	.	.	1		
	RM & YPS Resistant	RM	.	YPS	RM	.	YPS	0	3	
		.	.	YPS	RM	.	YPS	1		
		RM	.	.	RM	.	YPS	1		
		RM	.	YPS	.	.	YPS	0		
		RM	.	YPS	RM	.	.	1		
BY = YPS doubleton	BY & YPS Resistant	BY	BY	.	.	YPS	YPS	0	7	10
		.	BY	.	.	YPS	YPS	1		
		BY	.	.	.	YPS	YPS	2		
		BY	BY	.	.	.	YPS	1		
		BY	BY	.	.	YPS	.	3		
	RM & YJM Resistant	RM	YJM	.	.	RM	YJM	1	3	
		.	YJM	.	.	RM	YJM	0		
		RM	.	.	.	RM	YJM	0		
		RM	YJM	.	.	.	YJM	0		
		RM	YJM			RM		2		
