# Supplementary material for: Genetic Architecture of Highly Complex Chemical Resistance Traits across Four Yeast Strains
Source: PLoS Genet. 2012 Mar 15;8(3):e1002570. doi: 10.1371/journal.pgen.1002570 (PMC3305394; doi:10.1371/journal.pgen.1002570)
Supplement: Supplementary file 16 [file pgen.1002570.s016.doc]

No. of alleles	Allelic series	Pattern of detection						Missing relationships	Counts	Class Total
		BYxRM	BYxYJM	BYxYPS	RMxYJM	RMxYPS	YJMxYPS			
4	BY > YJM > RM > YPS	BY	BY	BY	YJM	RM	YJM		2	8
	RM > BY > YPS > YJM	RM	BY	BY	RM	RM	YPS		1	
	RM > YPS > YJM > BY	RM	YJM	.	.	RM	YPS	RM > YJM, YPS > BY	1	
	YPS > BY > RM > YJM	BY	BY	YPS	RM	YPS	YPS		1	
	YPS > BY > YJM > RM	BY	BY	YPS	YJM	.	.	YPS > RM, YPS > YJM	1	
	YPS > RM > BY > YJM	RM	BY	YPS	RM	YPS	YPS		2	
≥ 3	BY = RM > YJM > YPS	.	BY	BY	RM	.	YJM	RM > YPS	1	30
	BY = RM > YPS > YJM	.	BY	BY	.	RM	YPS	RM > YJM	1	
	BY > RM = YJM > YPS	BY	.	BY	.	RM	YJM	BY > YJM	3	
	BY > YJM = YPS > RM	BY	BY	BY	YJM	YPS	.		1	
	BY > YJM = YPS > RM	BY	BY	.	YJM	YPS	.	BY > YPS	1	
	BY > YJM > RM = YPS	BY	BY	BY	YJM	.	YJM		1	
	BY>YJM=YPS>RM	BY	BY	BY	YJM	YPS	.		1	
	RM > YJM = YPS > BY	RM	YJM	YPS	RM	RM	.		1	
		RM	.	YPS	RM	RM	.	YJM > BY	1	
	RM > YJM > BY = YPS	RM	YJM	.	RM	RM	YJM		1	
	YJM = YPS > BY > RM	BY	YJM	YPS	.	YPS	.	YJM > RM	1	
		BY	YJM	.	YJM	YPS	.	YPS > BY	1	
	YJM > BY = YPS > RM	BY	YJM	.	YJM	YPS	YJM		1	
	YJM > RM = YPS > BY	RM	YJM	YPS	YJM	.	YJM		2	
	YPS > YJM > BY = RM	.	YJM	YPS	YJM	YPS	YPS		2	
	BY = RM > YPS > YJM or RM > BY = YPS > YJM	.	BY	.	RM	RM	YPS	BY > YPS or RM > BY	1	
	BY > RM = YJM > YPS or BY > RM > YJM = YPS	BY	BY	BY	.	RM	.	YJM > YPS or RM > YJM	1	
	BY > RM = YPS > YJM or BY > RM > YJM =YPS	BY	BY	BY	RM	.	.	YPS > YJM or RM > YPS	1	
	BY > YJM = YPS > RM or BY > YPS > RM = YJM	BY	BY	BY	.	YPS	.	YJM > RM or YPS > YJM	1	
	RM = YJM > YPS >BY or RM > YJM = YPS > BY	RM	YJM	YPS	.	.	YJM	RM > YPS or RM > YJM	1	
	RM > BY = YJM > YPS or RM > BY > YJM = YPS	RM	.	BY	RM	RM	.	YJM > YPS or BY > YJM	1	
	YJM = YPS > RM >BY or YPS > RM = YJM > BY	RM	YJM	YPS	.	YPS	.	YJM > RM or YPS > YJM	2	
	YJM > YPS = BY > RM or YJM > YPS > BY = RM	.	YJM	.	YJM	YPS	YJM	BY > RM or YPS > BY	1	
	YPS = BY > RM > YJM or YPS > BY = RM > YJM	.	BY	.	RM	YPS	YPS	BY > RM or YPS > BY	1	
	YPS > BY = RM > YJM or YPS > RM > BY = YJM	.	.	YPS	RM	YPS	YPS	BY > YJM or RM > BY	1	
